# Supplementary material for: HIVprotI: an integrated web based platform for prediction and design of HIV proteins inhibitors
Source: J Cheminform. 2018 Mar 9;10:12. doi: 10.1186/s13321-018-0266-y (PMC5845081; doi:10.1186/s13321-018-0266-y)
Supplement: Supplementary file 2 — Additional file 2. Source code of HIVProtI web server. [file 13321_2018_266_MOESM2_ESM.zip › HIVprotI_Source-code/hivprotI/weka-3-6-6/doc/weka/associations/CheckAssociator.html]

CheckAssociator


---


|  |  |  |
| --- | --- | --- |
| **Overview** \| **Package** \| **Class** \| **Tree** \| **Deprecated** \| **Index** \| **Help** \| **Weka's home** | |  |
| **PREV CLASS**   **NEXT CLASS** | **FRAMES**    **NO FRAMES**     **All Classes** |  |
| SUMMARY: NESTED \| FIELD \| CONSTR \| METHOD | DETAIL: FIELD \| CONSTR \| METHOD |  |


---


## weka.associations Class CheckAssociator

```
java.lang.Object
  weka.core.Check
      weka.core.CheckScheme
          weka.associations.CheckAssociator
```

**All Implemented Interfaces:**: OptionHandler, RevisionHandler

---

``` public class CheckAssociator extends CheckScheme implements RevisionHandler ```

Class for examining the capabilities and finding problems with
associators. If you implement an associators using the WEKA.libraries,
you should run the checks on it to ensure robustness and correct
operation. Passing all the tests of this object does not mean
bugs in the associators don't exist, but this will help find some
common ones.

Typical usage:

`java weka.associations.CheckAssociator -W associator_name
-- associator_options` 

CheckAssociator reports on the following:

- Associator abilities
  - Possible command line options to the associators
  - Whether the associators can predict nominal, numeric, string,
    date or relational class attributes.
  - Whether the associators can handle numeric predictor attributes
  - Whether the associators can handle nominal predictor attributes
  - Whether the associators can handle string predictor attributes
  - Whether the associators can handle date predictor attributes
  - Whether the associators can handle relational predictor attributes
  - Whether the associators can handle multi-instance data
  - Whether the associators can handle missing predictor values
  - Whether the associators can handle missing class values
  - Whether a nominal associators only handles 2 class problems
  - Whether the associators can handle instance weights
- Correct functioning
  - Correct initialisation during buildAssociations (i.e. no result
    changes when buildAssociations called repeatedly)
  - Whether the associators alters the data pased to it
    (number of instances, instance order, instance weights, etc)
- Degenerate cases
  - building associators with zero training instances
  - all but one predictor attribute values missing
  - all predictor attribute values missing
  - all but one class values missing
  - all class values missing

Running CheckAssociator with the debug option set will output the
training dataset for any failed tests.

The `weka.associations.AbstractAssociatorTest` uses this
class to test all the associators. Any changes here, have to be
checked in that abstract test class, too.


Valid options are:

```
 -D
  Turn on debugging output.
```

```
 -S
  Silent mode - prints nothing to stdout.
```

```
 -N <num>
  The number of instances in the datasets (default 20).
```

```
 -nominal <num>
  The number of nominal attributes (default 2).
```

```
 -nominal-values <num>
  The number of values for nominal attributes (default 1).
```

```
 -numeric <num>
  The number of numeric attributes (default 1).
```

```
 -string <num>
  The number of string attributes (default 1).
```

```
 -date <num>
  The number of date attributes (default 1).
```

```
 -relational <num>
  The number of relational attributes (default 1).
```

```
 -num-instances-relational <num>
  The number of instances in relational/bag attributes (default 10).
```

```
 -words <comma-separated-list>
  The words to use in string attributes.
```

```
 -word-separators <chars>
  The word separators to use in string attributes.
```

```
 -W
  Full name of the associator analysed.
  eg: weka.associations.Apriori
  (default weka.associations.Apriori)
```

```
 Options specific to associator weka.associations.Apriori:
```

```
 -N <required number of rules output>
  The required number of rules. (default = 10)
```

```
 -T <0=confidence | 1=lift | 2=leverage | 3=Conviction>
  The metric type by which to rank rules. (default = confidence)
```

```
 -C <minimum metric score of a rule>
  The minimum confidence of a rule. (default = 0.9)
```

```
 -D <delta for minimum support>
  The delta by which the minimum support is decreased in
  each iteration. (default = 0.05)
```

```
 -U <upper bound for minimum support>
  Upper bound for minimum support. (default = 1.0)
```

```
 -M <lower bound for minimum support>
  The lower bound for the minimum support. (default = 0.1)
```

```
 -S <significance level>
  If used, rules are tested for significance at
  the given level. Slower. (default = no significance testing)
```

```
 -I
  If set the itemsets found are also output. (default = no)
```

```
 -R
  Remove columns that contain all missing values (default = no)
```

```
 -V
  Report progress iteratively. (default = no)
```

```
 -A
  If set class association rules are mined. (default = no)
```

```
 -c <the class index>
  The class index. (default = last)
```

Options after -- are passed to the designated associator.

**Version:**
:   $Revision: 1.7 $

**Author:**
:   Len Trigg (trigg@cs.waikato.ac.nz), FracPete (fracpete at waikato dot ac dot nz)

**See Also:**: `TestInstances`

---

**Nested Class Summary**

**Nested classes/interfaces inherited from class weka.core.CheckScheme**

`CheckScheme.PostProcessor`


| **Field Summary** | |
| --- | --- |
| `static int` | `NO_CLASS`             a "dummy" class type |


**Constructor Summary**

`CheckAssociator()`


| **Method Summary** | |
| --- | --- |
| `void` | `doTests()`             Begin the tests, reporting results to System.out |
| `Associator` | `getAssociator()`             Get the associator being tested |
| `java.lang.String[]` | `getOptions()`             Gets the current settings of the CheckAssociator. |
| `java.lang.String` | `getRevision()`             Returns the revision string. |
| `java.util.Enumeration` | `listOptions()`             Returns an enumeration describing the available options. |
| `static void` | `main(java.lang.String[] args)`             Test method for this class |
| `void` | `setAssociator(Associator newAssociator)`             Set the associator to test. |
| `void` | `setOptions(java.lang.String[] options)`             Parses a given list of options. |

**Methods inherited from class weka.core.CheckScheme**

`attributeTypeToString, getNumDate, getNumInstances, getNumInstancesRelational, getNumNominal, getNumNumeric, getNumRelational, getNumString, getPostProcessor, getWords, getWordSeparators, hasClasspathProblems, setNumDate, setNumInstances, setNumInstancesRelational, setNumNominal, setNumNumeric, setNumRelational, setNumString, setPostProcessor, setWords, setWordSeparators`

**Methods inherited from class weka.core.Check**

`getDebug, getSilent, setDebug, setSilent`

**Methods inherited from class java.lang.Object**

`equals, getClass, hashCode, notify, notifyAll, toString, wait, wait, wait`

**Field Detail**

### NO\_CLASS

```
public static final int NO_CLASS
```

:   a "dummy" class type

    **See Also:**: Constant Field Values


**Constructor Detail**

### CheckAssociator

```
public CheckAssociator()
```


**Method Detail**

### listOptions

```
public java.util.Enumeration listOptions()
```

:   Returns an enumeration describing the available options.

    :   **Specified by:**: `listOptions` in interface `OptionHandler` **Overrides:**: `listOptions` in class `CheckScheme`
    :   **Returns:**: an enumeration of all the available options.

---


### setOptions

```
public void setOptions(java.lang.String[] options)
                throws java.lang.Exception
```

:   Parses a given list of options.
    Valid options are:

    ```
     -D
      Turn on debugging output.
    ```

    ```
     -S
      Silent mode - prints nothing to stdout.
    ```

    ```
     -N <num>
      The number of instances in the datasets (default 20).
    ```

    ```
     -nominal <num>
      The number of nominal attributes (default 2).
    ```

    ```
     -nominal-values <num>
      The number of values for nominal attributes (default 1).
    ```

    ```
     -numeric <num>
      The number of numeric attributes (default 1).
    ```

    ```
     -string <num>
      The number of string attributes (default 1).
    ```

    ```
     -date <num>
      The number of date attributes (default 1).
    ```

    ```
     -relational <num>
      The number of relational attributes (default 1).
    ```

    ```
     -num-instances-relational <num>
      The number of instances in relational/bag attributes (default 10).
    ```

    ```
     -words <comma-separated-list>
      The words to use in string attributes.
    ```

    ```
     -word-separators <chars>
      The word separators to use in string attributes.
    ```

    ```
     -W
      Full name of the associator analysed.
      eg: weka.associations.Apriori
      (default weka.associations.Apriori)
    ```

    ```
     Options specific to associator weka.associations.Apriori:
    ```

    ```
     -N <required number of rules output>
      The required number of rules. (default = 10)
    ```

    ```
     -T <0=confidence | 1=lift | 2=leverage | 3=Conviction>
      The metric type by which to rank rules. (default = confidence)
    ```

    ```
     -C <minimum metric score of a rule>
      The minimum confidence of a rule. (default = 0.9)
    ```

    ```
     -D <delta for minimum support>
      The delta by which the minimum support is decreased in
      each iteration. (default = 0.05)
    ```

    ```
     -U <upper bound for minimum support>
      Upper bound for minimum support. (default = 1.0)
    ```

    ```
     -M <lower bound for minimum support>
      The lower bound for the minimum support. (default = 0.1)
    ```

    ```
     -S <significance level>
      If used, rules are tested for significance at
      the given level. Slower. (default = no significance testing)
    ```

    ```
     -I
      If set the itemsets found are also output. (default = no)
    ```

    ```
     -R
      Remove columns that contain all missing values (default = no)
    ```

    ```
     -V
      Report progress iteratively. (default = no)
    ```

    ```
     -A
      If set class association rules are mined. (default = no)
    ```

    ```
     -c <the class index>
      The class index. (default = last)
    ```

    :   **Specified by:**: `setOptions` in interface `OptionHandler` **Overrides:**: `setOptions` in class `CheckScheme`
    :   **Parameters:**: `options` - the list of options as an array of strings **Throws:**: `java.lang.Exception` - if an option is not supported

---


### getOptions

```
public java.lang.String[] getOptions()
```

:   Gets the current settings of the CheckAssociator.

    :   **Specified by:**: `getOptions` in interface `OptionHandler` **Overrides:**: `getOptions` in class `CheckScheme`
    :   **Returns:**: an array of strings suitable for passing to setOptions

---


### doTests

```
public void doTests()
```

:   Begin the tests, reporting results to System.out

    :   **Specified by:**: `doTests` in class `CheckScheme`

---


### setAssociator

```
public void setAssociator(Associator newAssociator)
```

:   Set the associator to test.

    :   **Parameters:**: `newAssociator` - the Associator to use.

---


### getAssociator

```
public Associator getAssociator()
```

:   Get the associator being tested

    :   **Returns:**: the associator being tested

---


### getRevision

```
public java.lang.String getRevision()
```

:   Returns the revision string.

    :   **Specified by:**: `getRevision` in interface `RevisionHandler`
    :   **Returns:**: the revision

---


### main

```
public static void main(java.lang.String[] args)
```

:   Test method for this class

    :   **Parameters:**: `args` - the commandline parameters


---


|  |  |  |
| --- | --- | --- |
| **Overview** \| **Package** \| **Class** \| **Tree** \| **Deprecated** \| **Index** \| **Help** \| **Weka's home** | |  |
| **PREV CLASS**   **NEXT CLASS** | **FRAMES**    **NO FRAMES**     **All Classes** |  |
| SUMMARY: NESTED \| FIELD \| CONSTR \| METHOD | DETAIL: FIELD \| CONSTR \| METHOD |  |


---
